# Facial Emotion Recognition Impairment in Patients with Parkinson's Disease and Isolated Apathy

**DOI:** 10.4061/2010/930627

**Published:** 2010-07-28

**Authors:** Mercè Martínez-Corral, Javier Pagonabarraga, Gisela Llebaria, Berta Pascual-Sedano, Carmen García-Sánchez, Alexandre Gironell, Jaime Kulisevsky

**Affiliations:** Movement Disorders Unit, Neurology Department, Sant Pau Hospital, Sant Pau Institute of Biomedical Research (IIB-Sant Pau), Autonomous University of Barcelona and Centro de Investigación Biomédica en Red-Enfermedades Neurodegenerativas (CIBERNED), Sant Antoni M. Claret 167, 08025 Barcelona, Spain

## Abstract

Apathy is a frequent feature of Parkinson's disease (PD), usually related with executive dysfunction. However, in a subgroup of PD patients apathy may represent the only or predominant neuropsychiatric feature. To understand the mechanisms underlying apathy in PD, we investigated emotional processing in PD patients with and without apathy and in healthy controls (HC), assessed by a facial emotion recognition task (FERT). We excluded PD patients with cognitive impairment, depression, other affective disturbances and previous surgery for PD. PD patients with apathy scored significantly worse in the FERT, performing worse in fear, anger, and sadness recognition. No differences, however, were found between nonapathetic PD patients and HC. These findings suggest the existence of a disruption of emotional-affective processing in cognitive preserved PD patients with apathy. To identify specific dysfunction of limbic structures in PD, patients with isolated apathy may have therapeutic and prognostic implications.

## 1. Introduction

Apathy has been defined as a lack of motivation evidenced by diminished goal-directed behavior, cognition, and emotion [1]. Another definition, focusing on observable aspects, is a quantitative reduction of self-generated voluntary and purposeful behaviors [2]. 

Apathy is a frequent feature of Parkinson's disease (PD), with a prevalence ranging from 5% to 51% of PD patients [3–9]. Lower prevalence has been observed when patients with depression or dementia were excluded [8] whereas a higher prevalence has been reported in samples including other neuropsychiatric disturbances [6]. Although apathy and depression frequently coexist in PD, they can develop separately [5, 6]. Apathy has been consistently reported to be associated with executive dysfunction [4, 5, 7, 8, 10–12], suggesting that the dysfunction of frontal-subcortical circuits is common to both phenomena in PD [7, 13, 14]. Nevertheless, disruption of emotional-affective functional circuits seems also to be present since the early stages of the disease, and may play an additional role in the development of apathy in patients with otherwise no apparent cognitive deficits. 

Further insight into the pathophysiology of apathy in PD should come from studies of cognitively intact patients with apathy as the only or predominant neuropsychiatric feature. To the best of our knowledge, apathy has not been formally assessed in this PD population. More specifically, emotion processing has not been evaluated in apathetic PD patients. We aimed to explore emotion processing, as assessed by a facial emotion recognition task, in cognitively intact and nondepressed PD patients with and without apathy, matched for age and educational level. 

## 2. Methods

### 2.1. Subjects

PD patients fulfilling diagnostic criteria for PD [15] were prospectively recruited from our outpatient Movement Disorders Clinic. Patients with cognitive impairment, as diagnosed by a score ≥0,5 in the Clinical Dementia Rating Scale and a score >0 on the UPDRS-cognition item, were excluded. We excluded also patients with depression (DSM-IV criteria and a score ≥11 in the Hospital Anxiety and Depression Scale-HADS) [16], visuoperceptive impairment (Facial Recognition Test, short form version >20) [17], those with previous surgery for PD, and those taking drugs with possible interference in emotion recognition tests such as beta blockers [18]. Diagnosis of apathy was based on a clinical interview aiming to identify a reduction of self-generated voluntary and purposeful behaviors that was not merely due to the presence of parkinsonian motor symptoms, and on a score ≥2 on the motivation/initiative item of the mentation part of the UPDRS [19].

Twelve PD patients with apathy and 19 matched nonapathetic PD patients participated in the study after giving informed consent. All participants were at stable doses of dopaminergic drugs during the 4 weeks before inclusion. All PD patients were examined during the “on” state. Motor status and severity of the disease were assessed by the motor section of the Unified Parkinson's Disease Rating Scale (UPDRS-III) and Hoehn and Yahr Scale [20]. Global cognitive function was assessed using the Mattis Dementia Rating Scale (MDRS) [21].

Sixteen healthy control subjects (HC) participated in the study. They had no history of neurological or psychiatric disease and were comparable in age, gender, and education. No significant differences between groups were found in clinical or demographical variables (Table 1).

### 2.2. Facial Emotion Recognition Task

This task is based on a series of standardized pictures of faces showing six basic emotions: happiness, sadness, anger, fear, surprise, and disgust used as facial emotion recognition test (FERT) (JACFEE) [22]. Thirty-six pictures are included (6 for each emotion) and played by Caucasian and Oriental actors. These images were shown without any adaptation, and displayed on a computer screen (size 20 × 20 cm) in a well-illuminated room. Patients were comfortably seated at a distance of 45 cm of the screen. Subjects' task is to identify the emotion portrayed by each face. A brief explanation of each basic emotion (without showing any image) was done before starting the test. The faces were presented in random order. Each image was displayed during 3 seconds. After each image, a blank screen appeared and subjects had no time limit to respond. A list with the names of the six basic emotions was available, in case of word-finding difficulties. For all patients this was the first time receiving a facial emotion recognition test and no kind of feedback was given during the task. The percentage of errors committed in the identification of each emotion was recorded.

## 3. Statistical Analysis

Group differences in demographic, clinical, and cognitive characteristics were analyzed using a univariate analysis of variance and *χ* ^2^ when appropriated. A repeated measures analysis of variance (ANOVA) was used to test for an effect of group on the FERT performance. A post hoc analysis (Scheffe) was conducted to identify differences between particular subgroups. Associations between FERT scores and demographic, clinical, and cognitive variables were analyzed with Spearman's rank correlations. Variables showing a significant correlation with FERT performance were included as covariables in a second analysis. The significance level was set at 0.05 for all parameters.

## 4. Results

Results in the FERT are shown in Table 2. The performances in the FERT were compared using a repeated measures analysis of variance, with Huyn-Feldt correction for non-sphericity. Group (PD patients with apathy, PD patients without apathy, and healthy controls) was the between-subject factor, and emotion (happiness, surprise, fear, disgust, anger, and sadness) the within-subject factor. We observed both a group effect [*F*(2, 44) = 5.877, *P* = .005] and an emotion effect [*F*(4.263, 187.556) = 20.10, *P* < .001], with a significant interaction between group and emotion [*F*(8.525, 187.556) = 3.031, *P* = .003]. Post hoc analysis showed significant differences between apathetic PD patients and both nonapathetic patients (*P* = .012) and HC (*P* = .007). No significant differences were observed between nonapathetic PD patients and HC.

Although no significant differences between groups were found in clinical and demographical variables, apathetic PD patients tended to be older, less educated, and with lower scores in the MDRS, and they also tended to receive lower doses of dopaminergic treatment. In order to explore the possible influence of these variables in the FERT score (in both apathetic and nonapathetic PD patients), correlations between FERT global performance and age, years of education, MDRS score, and dopaminergic treatment doses were analyzed. Significant correlation was found with age (*r* = 0.312, *P* = .033), education (*r* = −0.295, *P* = .046), and MDRS (*r* = −0.455, *P* = .025). A significant group effect (*F* = 7.438, *P* = .013) was still observed after covariance by age, years of education, and MDRS. 

In order to explore whether emotional processing was homogeneous to all emotions or if the recognition of emotions selectively affected particular emotions, the analysis of the different emotions examined revealed that apathetic PD patients scored significantly worse only in the recognition of fear (*F* = 4.948, *P* = .012) anger (*F* = 4.393, *P* = .018), and sadness (*F* = 4.447, *P* = .018).

## 5. Discussion

The main results of the present study show that PD patients with apathy exhibit selective deficits in facial emotion recognition, with more specific deficits in the recognition of fear, anger, and sadness. Conversely, nonapathetic patients recognized facial emotions as accurately as healthy controls.

The exclusion of PD patients with some degree of cognitive impairment, along with the persistence of significant facial emotion recognition differences between groups after covariance for MDRS score, education, and age supports the hypothesis that the differences observed between apathetic and nonapathetic patients are not due to task complexities, but to a differential impairment of the brain circuits involved in emotion recognition. Moreover, when recognition of particular emotions was analyzed, the apathetic group of PD patients showed a different pattern of difficulties. As previously observed, not all basic emotions are as easy to identify. Fear is often mistaken with surprise, and disgust with anger [23]. In our sample, fear and disgust were the most difficult emotions to identify for nonapathetic PD patients and HC. However, while in apathetic patients fear recognition was clearly affected, disgust recognition was not impaired, and deficits in fear, sadness, and anger recognition were also observed. This finding reinforces the hypothesis that emotion recognition defects in apathetic PD patients are not generalized but restricted to particular emotions.

The use of the FERT precludes a precise identification of the physiopathological correlates of the emotional deficits observed in our sample. Although limbic structures are essential in emotion processing, and a link between the amygdala and fear recognition is well established [24, 25], facial emotion recognition involves multiple cerebral structures (visual, somatosensory, prefrontal-orbitofrontal and ventromedial-cortex, amygdala and basal ganglia) and also different neural processes (categorization of specific facial treats, and evocation of associated knowledge, generation of an emotional response in the observer) [23, 26, 27]. 

A remarkable observation of this study is the absence of significant differences between nonapathetic PD patients and HC in the facial emotion recognition task. Several studies explored explicit recognition of emotional information in PD. Overall, previous studies point towards the existence of deficits in emotion recognition [28–33] that can be partially reversed by dopaminergic medication [29, 32, 33], but conflicting results have been reported recently [34–36]. These differences, based on our results, could be due to the lack of control of the presence of apathy in the patients explored. While depression has been usually considered as a confounding factor when assessing the ability to recognize facial emotions, the lack of control of apathy severity could account for the different results observed in previous studies. 

Based on the literature, the lack of significant differences between nonapathetic PD patients and HC may be due to several causes. First, dopamine neurotransmission has been linked to the identification of emotions and we cannot exclude a beneficial effect of medication. For instance, Sprengelmeyer et al. compared facial emotion recognition between 16 untreated, early PD patients, 20 treated, more advanced PD patients, and 40 healthy controls [29]. Although facial emotion recognition was found to be impaired in both PD groups, deficits were more consistently noted in the nonmedicated group. Nonetheless, our results based on a sample of PD patients studied in their dopaminergic “on” state, agree with other studies [34, 36] which failed to identify significant evidence of facial emotion recognition impairment in medicated PD patients compared with HC. Second, the facial emotion expressions used in the present study were of high intensity. A variety of assessment procedures for the evaluation of facial emotion expressions recognition has been used, like expressions of different intensities [32] or tests created on purpose of a specific study [28, 30]. Suzuki et al. identified deficits in disgust recognition by means of a refined test and data analysis while no differences were identified with conventional measures. Third, our nonapathetic patients were not depressed and were free of any other neuropsychiatric symptom. Although the relation between depression and facial emotion recognition in former studies is rather inconclusive, it has been negatively related with the recognition of fear [28]. Finally, a small sample size and a brief FERT may have difficult identification of significant differences between nonapathetic PD patients and HC. 

Despite the exploratory character of this study, our results point towards a possible existence of a disruption of emotional-affective processing in cognitively intact PD patients with apathy. Further studies, assessing the early processing of emotional information, are needed to investigate the physiopathological correlates of the facial emotion recognition deficits observed in apathetic PD patients. To identify a specific dysfunction of limbic structures in PD patients with isolated apathy may have therapeutic and prognostic implications.

## Figures and Tables

**Table 1:** Demographic and clinical characteristics of subject groups.

Characteristics	Apathy group	Nonapathy group	Healthy controls	*P *value
	*n* = 12	*n* = 19	*n* = 16	
Age (years)	65.67 (4.96)	60.37 (9.38)	60.53 (12.93)	.299
Gender (%male)	83.33	68.42	50	.176
Education (years)	9.00 (2.45)	11.52 (5.19)	11.00 (6.29)	.349
PD duration (years)	4.83 (2.95)	7.26 (4.26)	—	.095
Hoehn-Yahr*	2.00 (.00)	2.03 (0.353)	—	.799
UPDRS motor*	20.58 (9.75)	20.77 (5.11)	—	.944
Motor fluctuations (%)	8.33	42.1	—	.085
LDopa eq. dose	699.72 (345.62)	907.56 (537.79)	—	.309
MDRS score	135.00 (3.71)	137.64 (6.147)	—	.240

Note that means and standard deviations (in parentheses). Hoehn-Yahr staging ranges from 0 to 6 (most severe); UPDRS motor: motor scale of Unified Parkinson's Disease Rating Scale. Difference between groups using univariate analysis of variance comparisons or *χ* ^2^ (gender and motor fluctuations distribution). *Scores obtained from PD patients when they were taking their dopaminergic medications.

**Table 2:** Errors (%) on the facial emotion recognition test.

	Apathy group	Nonapathy group	Healthy controls	*P *value
				(post hoc)
Happiness	13.89 (17.16)	4.38 (7.54)	8.89 (10.66)	.097 (0.12)
Surprise	23.61 (18.06)	22.81 (20.19)	25.55 (27.36)	.937 (0.96)
Fear	69.44 (28.28)	40.35 (25.04)	36.66 (34.62)	**.012 (.032) **
Disgust	34.72 (20.66)	55.26 (24.25)	46.66 (28.31)	.092 (0.14)
Anger	38.89 (22.84)	18.42 (19.95)	18.89 (18.75)	**.018 (.038) **
Sadness	56.94 (33.68)	26.31 (21.74)	35.55 (30.12)	**.018 (.031)**

Total errors	39.35 (7.57)	27.77 (10.55)	28.33 (10.61)	**.006 (.012) **

Note that means and standard deviations (in parentheses). Difference between groups using univariate analysis of variance comparisons. Post hoc: “*P*” values after adjusting for age. Education, and MDRS total score.
